# A spectrogram and local feature-assisted convolutional neural network for Amharic speech emotion identification

**DOI:** 10.1038/s41598-026-54784-7

**Published:** 2026-05-27

**Authors:** Yeshambel Asmare Mengist, Abrham Debasu Mengistu, Mulatu Yirga Beyene, Mikiyas Assefa Kassa, Getasew Asmare Mengist

**Affiliations:** 1 Department of Computer Science, Debark University, Debark, Ethiopia; 2 Department of Information Technology, Wollo University, KIoT, Kombolcha, Ethiopia; 3 Faculty of Computing, Bahir Dar Institute of Technology, Bahirdar University, Bahirdar, Ethiopia

**Keywords:** Speech emotion identification, Convolutional neural network, Spectrogram, Local features, Amharic speech, Deep learning, Computational biology and bioinformatics, Engineering, Mathematics and computing, Neuroscience

## Abstract

Speech Emotion Recognition (SER) plays a significant role in improving human–computer interaction and human–human communication. Nevertheless, speech emotion recognition in low-resource languages like Amharic is still a difficult task because of the lack of datasets and language diversity. In this paper, a Convolutional Neural Network (CNN)-based approach, which combines spectrogram features and local acoustic features such as Mel-Frequency Cepstral Coefficients, chroma, zero-crossing rate, energy, and pitch, is proposed for efficient Amharic speech emotion recognition. A dataset of 1650 three-second Amharic speech samples was created, and the samples were labeled with five emotional classes: anger, fear, happy, neutral, and sad. Advanced preprocessing methods such as spectral subtraction and wavelet denoising were used to improve the quality of the signals and speed up the training process. The experimental results show that the proposed CNN-based approach has a classification accuracy of 90%, which is better than the recurrent neural network-based approaches: Long Short-Term Memory with 58.48%, Bidirectional Long Short-Term Memory with 63.33%, and Gated Recurrent Unit with 40%, as well as the single-feature models: local acoustic features with 73% and spectrogram features with 79%. These results confirm that the integration of spectrogram and local acoustic features within a CNN architecture improves accuracy and efficiency in speech emotion recognition in low-resource languages, setting a standard for future Amharic SER research.

## Introduction

Speech is the most natural form of communication between humans, and it is the most commonly used form. It is an effective medium for both Human–Computer Interaction (HCI) and human–human interaction. Apart from the information contained in the speech itself, there is the information contained in the tone, energy, pitch, rhythm, and rate, all of which help to reveal the emotional state of the person speaking^1,2^. The recognition of the emotional state can help to improve the efficiency of applications such as call center analytics, virtual assistants, videoconferences, and assistive technology^3,4^.

SER has been widely explored in well-resourced languages using benchmark datasets such as Berlin Database of Emotional Speech (BDES), Danish Emotional Speech (DES), and Interactive Emotional Dyadic Motion Capture (IEMOCAP)^5,6^. The initial techniques in speech emotion recognition employed handcrafted acoustic features like MFCCs, Chroma, Short-Term Energy, Pitch, ZCR, etc., along with traditional classifiers like Support Vector Machines (SVM), Gaussian Mixture Models (GMM), Hidden Markov Models (HMM), etc.^7,8^. Though these techniques were found to perform adequately in controlled environments, their performance was often restricted by the feature representation capabilities in noisy environments^9,10^.

Recent advancements in the development of deep learning methods have significantly improved the performance of SER systems by enabling the automatic learning of discriminative representations directly from the speech signals or time–frequency representations^2,11^. The transformation of the speech signals into images in the form of spectrogram representations, such as Mel-spectrograms and scalograms, has enabled the application of image-based deep learning methods, particularly CNNs, for the effective representation of emotions in the spectral domain^6,12^. Moreover, the application of recurrent neural networks, including LSTM and GRU architectures, has gained popularity for the effective representation of emotions in the temporal domain, while the application of CNN and Transformer architectures has shown improved performance in terms of accuracy and robustness in SER systems^13,14^.

Despite all these developments, research in SER continues to be dominated by high-resource languages such as English, Mandarin, and German^1,2^. Conversely, less-resourced languages like Amharic are poorly represented in SER research due to the lack of annotated emotional speech data and the language complexities posed by its distinctive phonetic, prosodic, and morphological characteristics^15,16^. Most existing research on African and less-resourced languages uses small data sets and traditional machine learning approaches, which are not only limited in terms of generalization performance but also yield poor accuracy in recognizing emotions^3,12^.

To address these challenges, a CNN-based SER framework has been proposed, specifically designed for Amharic, utilizing a combination of spectrogram features and other complementary local acoustic characteristics, such as MFCC, chroma, ZCR, energy, and pitch. The idea of feature fusion, where a combination of global feature sets and local feature sets is used, has been proven to improve robustness and classification performance, considering the complementary nature of feature spaces^9,17^. Furthermore, sophisticated preprocessing techniques such as spectral subtraction and wavelet transformation are utilized to address noise, a critical factor in feature quality, especially in real-world scenarios^3,15^.

This work presents several important contributions to the Amharic SER community, tackling issues related to low-resource languages. An annotated Amharic emotional speech dataset was created, consisting of five different classes of emotions: anger, fear, happy, neutral, and sad, which is a great addition to the community. A novel hybrid feature extraction approach was proposed, combining spectrogram-based visual features with traditional local acoustic features such as MFCC, chroma, pitch, energy, and ZCR, which efficiently captured both global and detailed emotional information. A CNN model optimized for the fused feature space was designed, outperforming traditional recurrent models such as LSTM, BiLSTM, and GRU networks. The experimental results validated the efficacy of the proposed solution, reporting high classification accuracy with well-balanced precision, recall, and F1-measures, which is a clear sign of excellent generalization capabilities. Finally, this work sets a new baseline for Amharic speech emotion identification and points out the possible real-world applicability of the proposed system in human–computer interaction, assistive systems, and communication-related applications.

This research makes significant progress in SER for low-resource languages and helps to fill the gap between high-resource and low-resource language technologies. In addition, it provides avenues for future studies in cross-lingual adaptation, multimodal emotion recognition, and inclusive AI systems for linguistic diversity. Apart from the technical aspects, this research has real-world implications for enhancing accessibility and communication in Amharic-speaking societies and helps to achieve the overall objective of indigenous language integration into modern intelligent systems.

## Related work

SER is one of the major research areas within the field of affective computing, which seeks to recognize the emotional states conveyed by the speech signal to enhance the performance of human–computer interfaces, healthcare applications, and intelligent services^18,19^. In the past, SER techniques mainly employed conventional machine learning techniques such as SVM, k-Nearest Neighbors (k-NN), GMM, HMM, along with manually designed acoustic features such as MFCC, Pitch, Energy, Chroma, ZCR^1,20^. Although the performance of the conventional SER techniques was satisfactory, the techniques failed to provide robust performance due to the limitations associated with the manually designed features.

The recent development of deep learning has greatly improved the SER task by allowing the automatic learning of features from the raw or lightly processed speech signal. The use of CNNs has been popular for learning spatial features from time–frequency maps like Mel-spectrograms, while Recurrent Neural Networks (RNNs), specifically LSTM and GRU networks, have been effective in modeling temporal relationships in emotional speech utterances^11,21^. The combination of CNNs and RNNs has further improved SER by modeling both spectral and temporal features, often performing better than separate models on standard datasets like IEMOCAP and Ryerson Audio-Visual Database of Emotional Speech and Song (RAVDESS)^14,22^.

Recent works have also investigated attention mechanisms and transformer architectures to overcome the shortcomings of traditional deep learning models. Transformers and CNN-Transformer models have shown remarkable effectiveness in modeling long-range contextual dependencies and global emotional patterns in speech, achieving competitive or state-of-the-art performance compared to traditional CNN-LSTM architectures^23,24^. Multi-scale feature learning and channel attention techniques, like squeeze-and-excitation modules, have also enhanced the robustness of SER models by focusing on emotionally discriminative spectral regions^22,25^.

Spectrogram-based features, especially Mel-spectrograms and wavelet scalograms, continue to be the most popular inputs for deep learning-based SER systems because of their capacity to effectively capture both temporal and spectral information^11,21^. Moreover, approaches involving feature fusion techniques that incorporate both global spectrogram features and local acoustic features, such as MFCCs, chroma, pitch, and energy, have been demonstrated to improve classification performance and generalization capabilities^26,27^. These feature fusion techniques exploit information from multiple feature spaces and have emerged as a prominent area of research in contemporary SER studies.

Despite the significant advances, most SER research efforts are confined to high-resource languages like English, German, and Mandarin, with large amounts of annotated data available^18,28^. In contrast, low-resource languages, especially African languages, are grossly underrepresented due to a lack of labeled data and their unique phonetic and prosodic properties. Amharic, one of the most widely spoken languages in Ethiopia, has its own set of challenges for SER, including complex morphology, syllable patterns, and intonation contours that are vastly different from Indo-European languages^29^.

Few recent studies have explored SER for the Amharic language^29^. Proposed the Amharic Speech Emotion Dataset (ASED) and presented the first benchmark for Amharic SER, exploring the effectiveness of CNNs for emotion classification. Later research by^30^ explored cross-corpus and multilingual SER with Amharic, observing significant performance drops when high-resource language models are used for Amharic, thus pointing out the importance of language-specific modeling approaches. Other studies on low-resource SER also confirm decreased accuracy and poor generalization when small datasets and traditional machine learning methods are employed^31^.

Recent reviews strongly suggest that effective preprocessing, feature fusion, and model optimization are essential for SER performance in low-resource conditions^19,21^. These observations call for the design of specialized SER systems that incorporate different feature sets and sophisticated noise removal methods.

To address these issues, this study proposes a CNN-based SER system tailored for Amharic speech. By incorporating spectrogram features with local acoustic characteristics and using optimized preprocessing methods, the proposed system is expected to enhance the classification accuracy and robustness in the low-resource scenario, thus making a contribution to the promotion of SER research for Amharic and other low-prevalent languages.

## Methodology

**Fig. 1:**
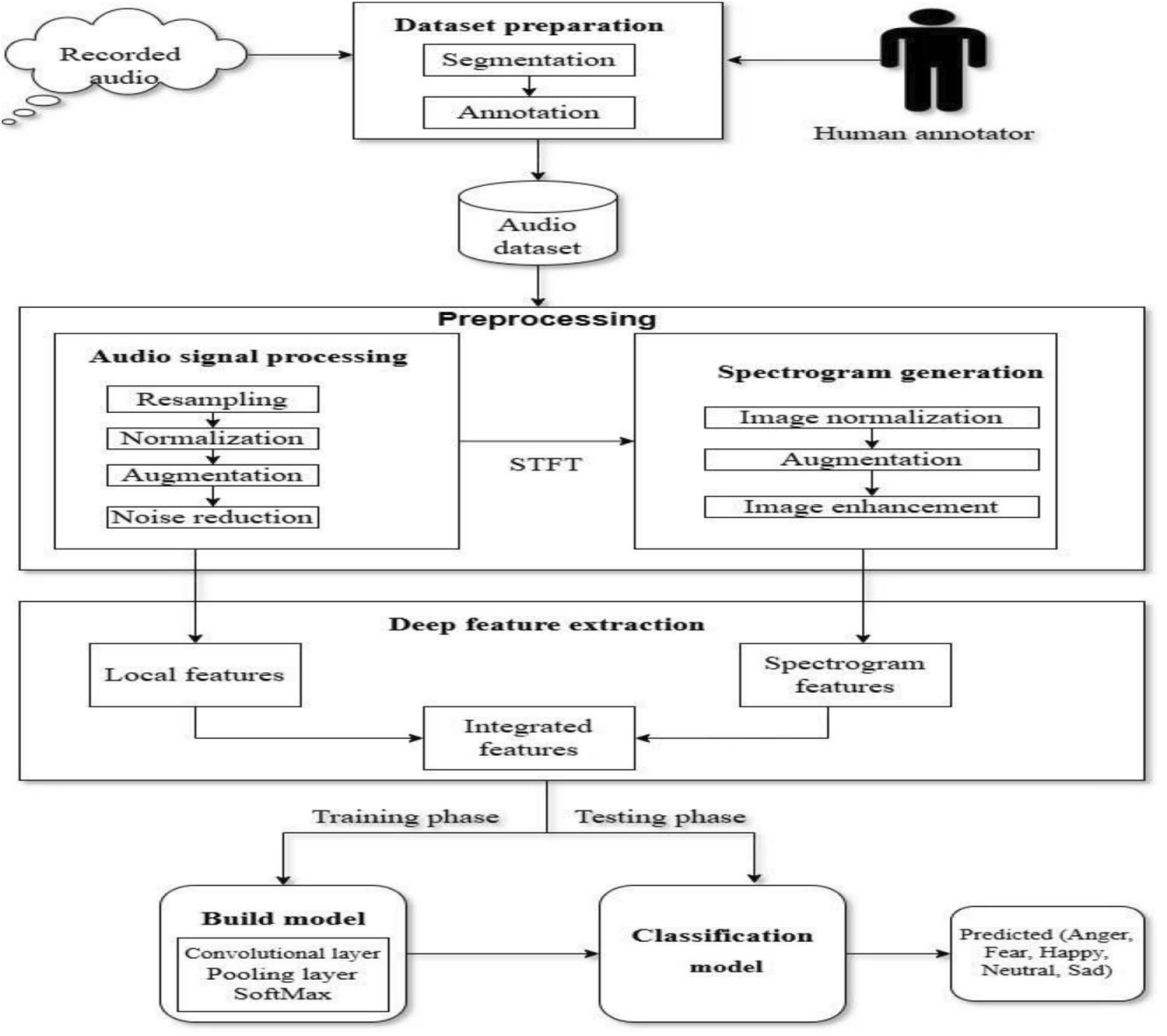
System architecture of the proposed Amharic SEI model.

The detailed methodology employed in the development of the proposed Amharic Speech Emotion Identification (SEI) system is presented. The system architecture, as shown in Fig. 1, is composed of four main stages: data acquisition, preprocessing, feature extraction, and emotion classification. In recent years, it is widely acknowledged that multi-stage pipelines are employed in the development of SER systems to enhance robustness and generalization capability, as supported by^18^ and^19^. In the proposed system, raw speech signals were collected from various real-world environments, such as homes, cafés, hospitals, and universities, to ensure the robustness of the proposed system, as shown in the work of^1^.

### Data collection

The dataset was built through a community-based participatory research design. The speech recordings were collected through smartphone devices with Infinix Hot 12 Pro and Tecno Spark 5. Smartphone data collection has been popular in recent research on SER studies. This is because smartphone data collection is accessible and allows researchers to collect speech data in a natural environment^20^. The dataset consists of speech samples obtained from approximately 35 speakers, including 19 males and 16 females, with ages varying from 21 to 36 years. The participants were chosen from a variety of demographic backgrounds. Recordings were collected from diverse real-world environments, such as homes, cafés, hospitals, and university, ensuring acoustic variety and strengthening the dataset’s robustness. The speech recordings were independently annotated by three experts from Debark University, including Amharic language specialists and psychology professionals. Each annotator labeled the recordings into five emotional categories: anger, fear, happy, neutral, and sad. Informed consent was obtained from all experts prior to their participation in the annotation process. Manual annotation by an expert is considered the best method to label emotions in a dataset, especially in a language that is not well resourced^29^. To make sure the annotations were reliable, a majority voting strategy was used to choose the final label for each sample. To avoid bias and make sure the evaluation was strong, a speaker-independent data split was used. This meant that speech samples from the same speaker were not used in both the training and testing sets. This method prevents data leakage and gives a more accurate picture of how well the model works in general. The final dataset is well-balanced with 330 samples in each emotion class. A well-balanced dataset is known to mitigate classification bias and stability in model training^14^.

### Ethics statement

This study involved human participants for speech data collection. All methods were carried out in accordance with relevant ethical guidelines and regulations. The research protocol was reviewed and approved by the Institutional Research Ethics Committee of Debark University, Ethiopia (Ref.: DKU/101/2025). Prior to participation, all subjects were informed about the objectives of the study and provided written informed consent for their speech recordings to be used for academic research purposes. Personal identities were not recorded, and all data were anonymized to ensure participant confidentiality.

### Data preprocessing

Audio files that were initially recorded in MP3 format were converted into WAV format to maintain signal quality and compatibility with available libraries for signal processing. The sampling frequency of the signals was standardized to 44.1 kHz. In addition, the stereo signals were converted into mono signals to maintain consistency in processing. The speech signals were segmented into fixed-length 3-s signals. This time length is commonly used in SER analysis to balance time context and processing efficiency^26^.

Furthermore, to minimize processing complexity and align with standard speech processing practices, all audio signals were resampled to 16 kHz sampling frequency, as recommended by^18^. In addition, amplitude normalization was performed to minimize the impact of recording variations resulting from different devices and environments^1^.

Data augmentation methods were used to improve the robustness of the proposed approach. These include adding background noise to the signals, time stretching, and pitch adjustment. These methods have been proven to be effective in improving the performance of the SER approach significantly^25,27^.

**Fig. 2:**
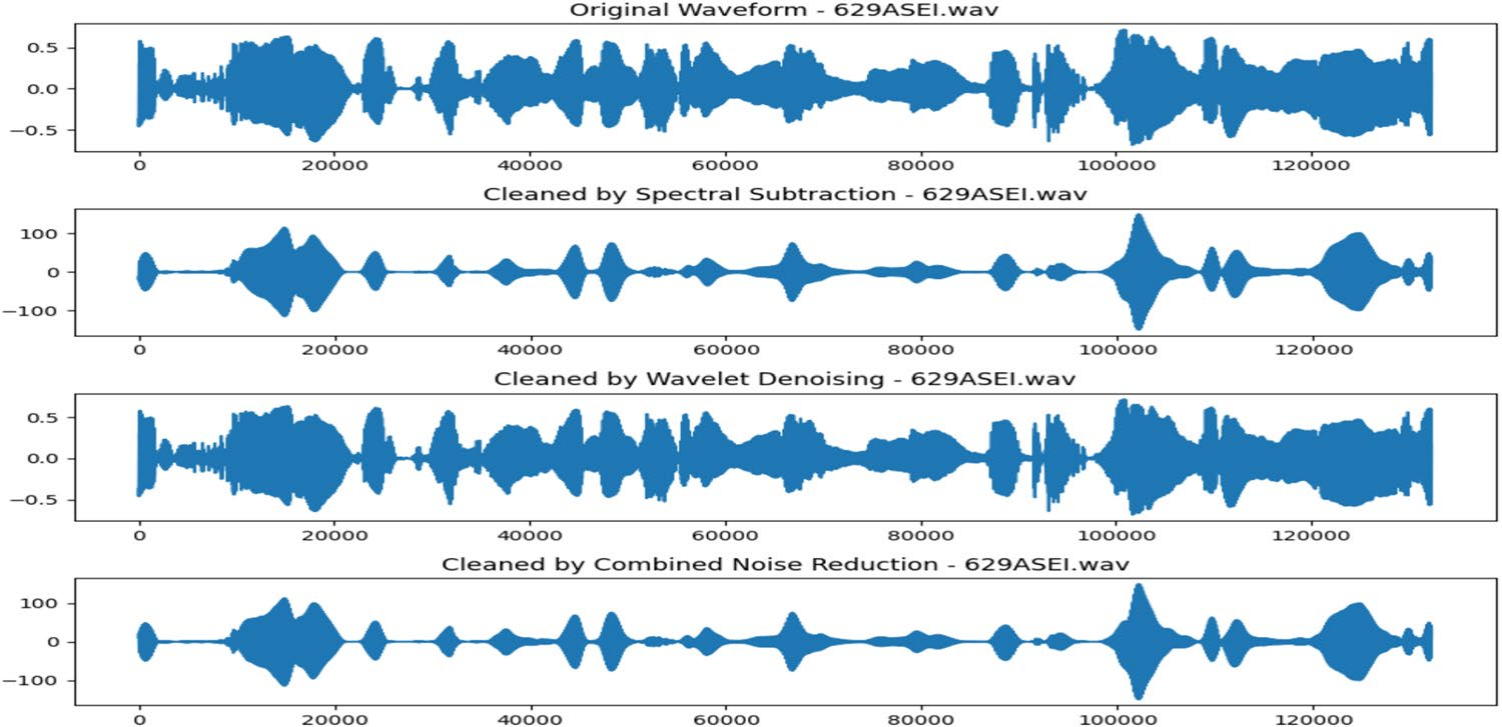
Sample waveform images illustrating the effect of each noise reduction method.

Noise reduction was achieved by using three different approaches: spectral subtraction, wavelet denoising, and a combined sequential approach. Spectral subtraction is a popular noise reduction technique for stationary noise, but it is less effective for non-stationary noise. Wavelet denoising is an effective technique for non-stationary noise, where noise is analyzed at different resolutions^32,33^. A combined approach is also proposed, where the advantages of both noise reduction techniques are utilized, and it is found to be effective for improving the intelligibility of speech in harsh acoustic environments^1^. The noise reduction approaches are shown in Fig. 2.

### Spectrogram generation and enhancement

The preprocessed audio signals were then converted into their respective time–frequency representations through the application of the Short-Time Fourier Transform (STFT) algorithm. Spectrograms were created by specifying the window size as 1024, hop size as 512, 128 Mel filter banks, and sampling rate as 16 kHz. Mel-spectrograms have been extensively employed in SER due to their perceptual relevance and suitability for CNN-based frameworks^11,21^.

The image preprocessing techniques employed for image enhancement were found to enhance the visibility of the spectral features and improve the efficiency of the CNN model’s training procedure. Image enhancement techniques have been found effective in enhancing classification accuracy when the spectrograms were treated as visual inputs^22^.

### Feature extraction

Two sets of complementary features were extracted to effectively represent both global and local emotional information:

- *Spectrogram features* The CNN architecture enables automatic hierarchical learning of spectral-temporal features from Mel-spectrogram images, which represent emotion-related patterns at various levels of abstraction^28^.
- *Local acoustic features*: Conventional acoustic features, such as MFCCs, chroma, zero-crossing rate (ZCR), energy, and pitch, were extracted using conventional signal processing methods. These features are highly effective in representing prosodic and phonetic properties related to emotional expression^26^.

**Fig. 3:**
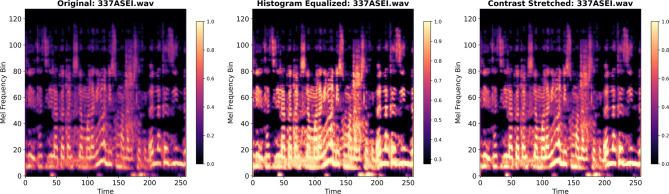
Spectrogram enhancement examples.

The two sets of extracted features were combined and processed together using the CNN architecture (Fig. 3). Fusion of features has proved to be an effective approach to enhance SER performance by exploiting complementary information from different feature spaces^22,27^.

### Model architecture

**Fig. 4:**
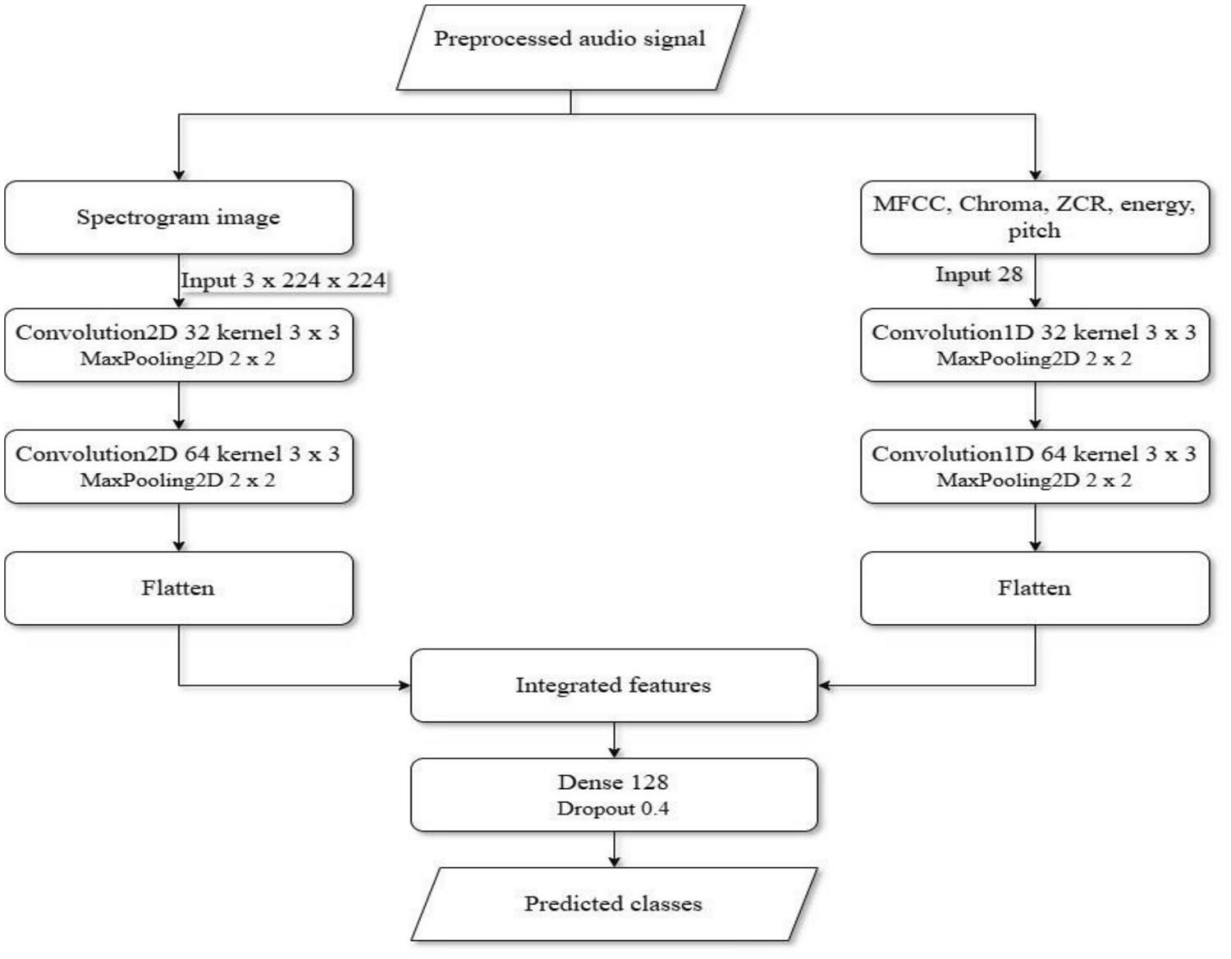
CNN architecture integrating spectrogram and local acoustic features.

The proposed CNN architecture in Fig. 4 is intended to learn both global and local representations of speech signals for recognizing emotions. CNN-based models are well-known for being good at spectrogram-based SER tasks because they can find spatial patterns in time–frequency representations^14,20^.

The proposed model combines global spectrogram representations with local handcrafted acoustic features to improve emotion discrimination, especially for low-resource languages like Amharic. This hybrid feature fusion method enables the model to capture both high-level spectral information and fine-grained acoustic features, which improves classification performance^29^.

Figure 4 shows that the architecture has two parallel branches. The first branch works with spectrogram images that come from audio signals that have already been processed. The input size is 224 × 224. It uses stacked convolutional layers with ReLU activation functions and max-pooling operations to get hierarchical spatial features while lowering the number of dimensions. The second branch works with handcrafted acoustic features like MFCC, Chroma, ZCR, energy, and pitch. To capture local temporal patterns, these features are modeled using one-dimensional convolutional layers with ReLU activation and pooling operations (Tables 1 and 2).

**Table 1:** Comparison of noise reduction techniques on accuracy and training time.

Noise reduction technique	Accuracy (%)	Training time (minutes: seconds)
Spectral Subtraction	86	16:44
Wavelet Transformation	83	18:32
Combined (Spectral + Wavelet)	90	12:11

**Table 2:** Effect of spectrogram image size on accuracy and training time.

Spectrogram image size	Accuracy (%)	Training time (minutes: seconds)
64 × 64	88	2:27
128 × 128	89	6:34
224 × 224	90	16:44
256 × 256	90	23:53

The size of 224 × 224 provided the best compromise between the two factors.

Both branches outputs are combined to generate a unified feature representation. Subsequently, a fully-connected layer consisting of 128 neurons and a dropout layer having a probability of 0.4 are applied in order to prevent overfitting, and then an output layer that predicts the predefined emotions is generated.

All models, namely CNN, LSTM, BiLSTM, and GRU, were evaluated for fairness using identical settings, such as dataset, preprocessing, and feature inputs. In addition, the RNN models were trained for either one or two layers and 128 hidden units each, while dropout was applied to reduce overfitting.

All models have been trained under similar settings and with optimized parameters, which have been listed in Table 3 below. During the training process, the optimizer used was Adam, whose learning rate was set to 0.0007, while the batch size was 32. The activation functions used were ReLU in all layers, while max pooling was utilized for dimensionality reduction. Dropout was set at 0.4 to help in generalization. Early stopping strategy was utilized to avoid overfitting, where the patience was 2. An image size of 224 × 224 of the spectrogram was utilized as the input, while noise reduction was carried out through a combination of techniques. This ensures that any differences in the performances of the models is due to differences in their architecture.

**Table 3:** Summaries of hyperparameters used for our experimental setups.

Parameter	Best choice
Pooling	MaxPooling
Optimizer	Adam
Batch Size	32
Activation	ReLU
Dropout Factor	0.4
Patience (early stop)	2
Learning Rate	0.0007
Spectrogram Size	224 × 224
Noise Reduction	Combined

### Evaluation metrics

To ensure a robust and unbiased evaluation, the dataset was divided into training (80%) and testing (20%) sets using a speaker-independent splitting strategy. This approach ensures that speech samples from the same speaker do not appear in both the training and testing sets, thereby preventing data leakage and avoiding artificially inflated performance. Consequently, it provides a more realistic assessment of the model’s ability to generalize to unseen speakers. Although a single train–test split was employed in this study, it offers a practical evaluation scenario for speaker-independent emotion recognition. However, future work will incorporate k-fold cross-validation to further enhance the robustness and reliability of the evaluation process.

Performance of the model was assessed by using the typical classification metrics such as accuracy, precision, recall, and F1-score. Each metric captures a different aspect of model performance. Accuracy helps to assess the general correctness of the algorithm, precision and recall allow measuring how effective the algorithm is in dealing with relevant data. F1-score is especially useful due to being a combination of the precision and recall rates. Confusion matrices were also used for assessing the performance on the level of separate classes. In particular, confusion matrices provide detailed insight into class-wise misclassification patterns that help us to gain a better understanding of the model performance in recognizing specific emotions. These metrics were widely used within SER research to provide the consistent model evaluation process^18,19^.

## Results and discussion

The experimental assessment of the proposed Amharic SEI system was performed on a dataset of 1650 speech samples for five emotional classes: anger, fear, happy, neutral, and sad. This study explores the effect of different feature representations, such as local acoustic features, spectrogram-based features, and their combination, on the performance of the system. The proposed CNN model was compared with conventional recurrent neural network models, such as LSTM, BiLSTM, and GRU networks, to evaluate the efficacy, robustness, and generalizability of the method for a low-resource language scenario.

### Experimental setup

All experiments were conducted using the Keras library for deep learning with TensorFlow as the backend. The system was deployed on a Windows 10 machine with a 64-bit architecture, an Intel Core i7-7500U processor running at 2.70 GHz, and 8 GB of RAM. The experiments were conducted using Python 3.9.0 and implemented within the Visual Studio Code environment, version 1.87.2. The audio processing and feature extraction were conducted using the Librosa library. The experimental environment was maintained consistently for all experiments with all models to ensure a fair comparison.

### Hyperparameter optimization experiments

#### Train-test split ratio

Four different ratios for splitting the data were used for evaluation, i.e., 70/30, 75/25, 80/20, and 85/15. The highest accuracy (90%) was achieved with the 80/20 split ratio; therefore, it was selected for all subsequent experiments.

#### Spectrogram parameter optimization

The parameters for the spectrogram were adjusted to ensure the extraction of detailed emotional features with computational efficiency. The FFT size, hop size, and number of Mel bands were set to 1024, 512, and 128, respectively. The Hamming window function was used to minimize leakage in the spectrum. Normalization ensured the features were consistent across the samples.

#### Audio noise reduction techniques

Three noise reduction methods, spectral subtraction, wavelet transformation, and a combination of both methods, were tested. From Table 1, it is evident that the combined noise reduction method (spectral subtraction and wavelet transformation) achieved the best performance in terms of accuracy (90%) while also reducing training time compared to the individual methods.

#### Spectrogram image resizing

The size of the spectrogram images was varied from 64 × 64 to 256 × 256. Although larger images resulted in higher accuracy, the training time also increased substantially. A resolution of 224 × 224 was chosen as it offered a good balance between accuracy and training time.

#### Hidden layers and activation functions

**Fig. 5:**
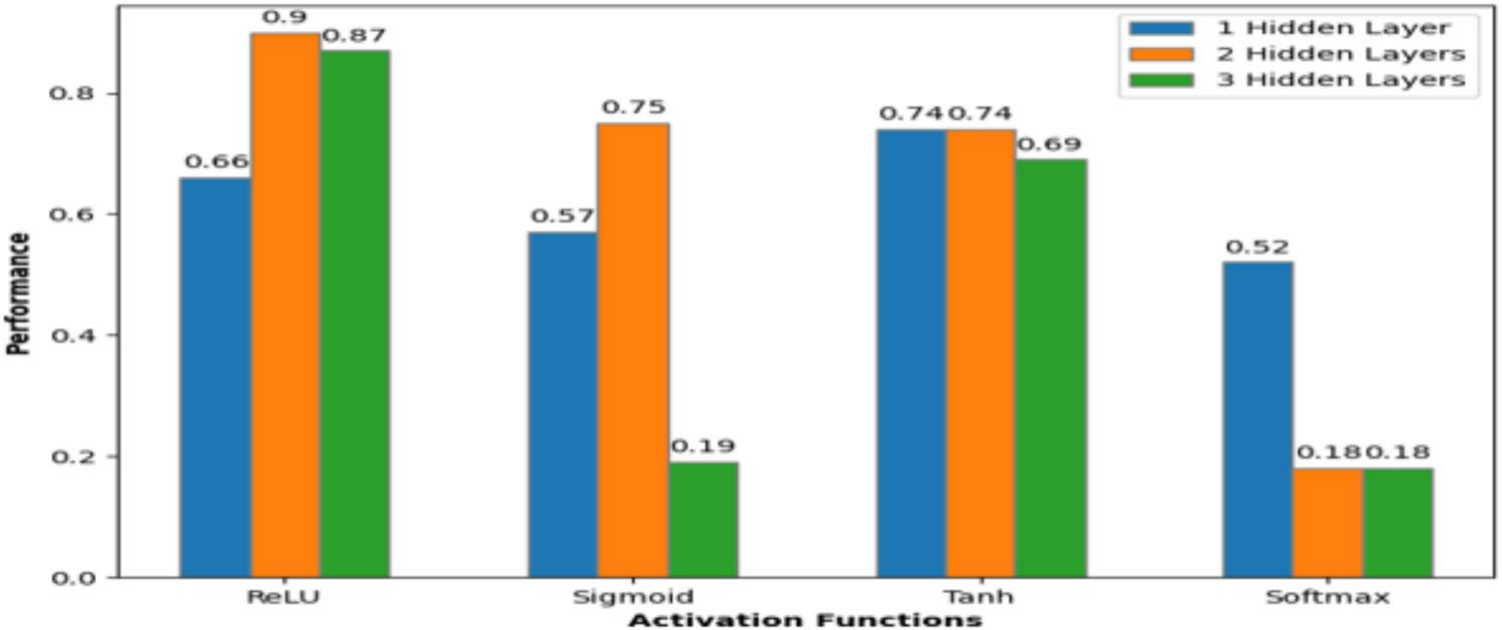
Accuracy comparison of different activation functions and hidden layer configurations.

The experiments were conducted to test different combinations of hidden layers. The best results were obtained by using two hidden layers with a ReLU activation function that produced 90% accuracy as shown in Fig. 5. The reason that ReLU is best suited to this problem is that it helps to solve the vanishing gradient problem.

#### Pooling and optimizer selection

MaxPooling and AveragePooling layers were also tested with optimizers such as SGD, Adam, RMSprop, and AdaGrad. MaxPooling with Adam optimizer had the highest accuracy of 90%, which justifies why it was chosen for the final model design.

#### Comparison of feature types

**Fig. 6:**
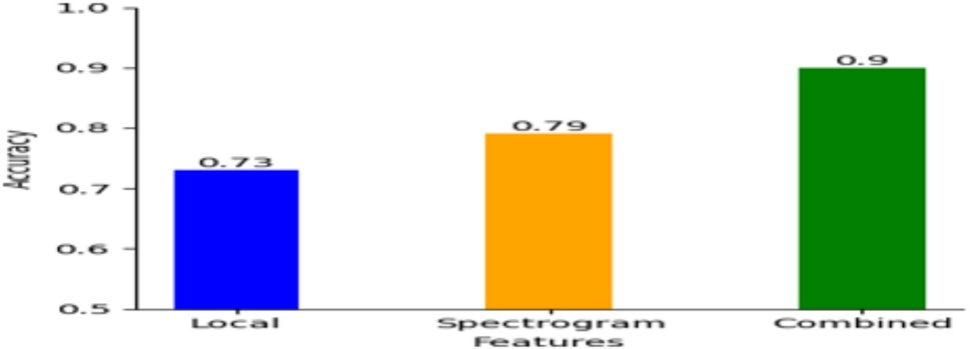
Classification accuracy using different feature types: local acoustic features, spectrogram features, and their integration.

Figure 6 illustrates the classification accuracy of local features only at 73%, spectrogram features only at 79%, and a combination of local and spectrogram features at 90%. The complementary nature of the two feature types significantly contributed to the improved classification accuracy, allowing for the distinction of subtle emotional expressions in Amharic speech (Table 4).

**Table 4:** Performance comparison of deep learning models for amharic speech emotion identification.

Model	Accuracy (%)	Precision	Recall	F1-Score
LSTM	58.48	0.60	0.58	0.59
BiLSTM	63.33	0.63	0.63	0.63
GRU	40.00	0.31	0.38	0.32
CNN	90.00	0.90	0.90	0.90

### Experimental results

#### LSTM model

**Fig. 7:**
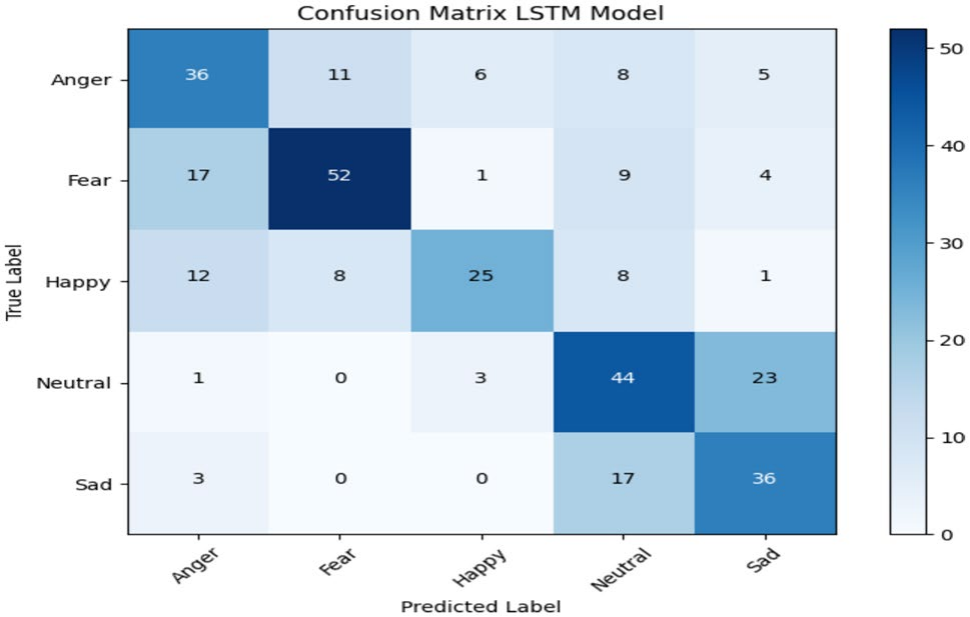
Confusion matrix of the LSTM model showing misclassifications between Neutral and Sad emotions.

**Fig. 8:**
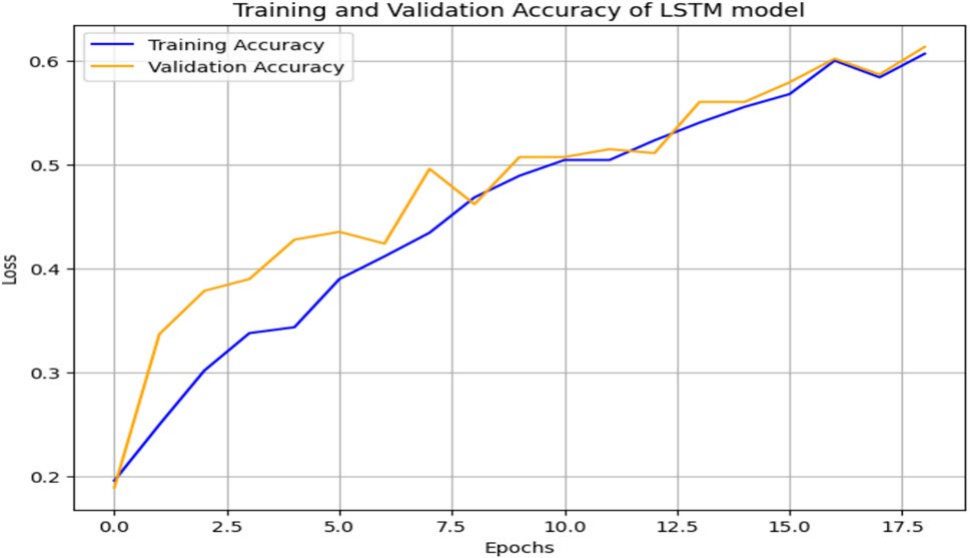
Training and validation accuracy of LSTM model.

**Fig. 9:**
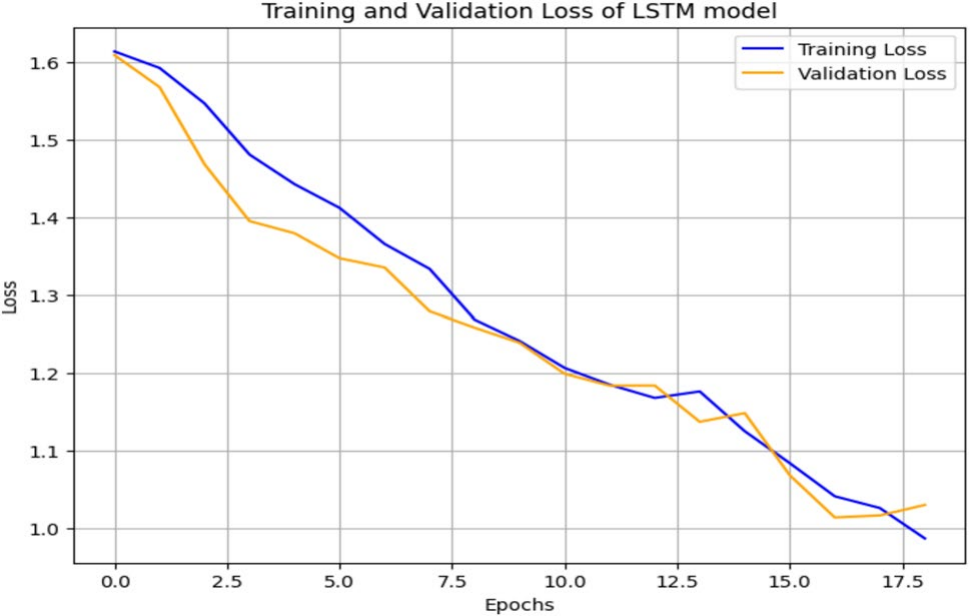
Training and validation loss of LSTM model.

The accuracy obtained by the sequential deep neural network model, i.e., the LSTM model, was 58.48%. The model was found to have reasonable values for precision and recall for the Fear emotion, but it was not effective for Happy and Neutral emotions. The confusion matrix in Fig. 7 indicated poor classification for the model, especially between Neutral and Sad emotions. The training and validation accuracy and loss curves, shown in Figs. 8 and 9, indicate stable learning behavior with minimal signs of overfitting.

#### BiLSTM model

**Fig. 10:**
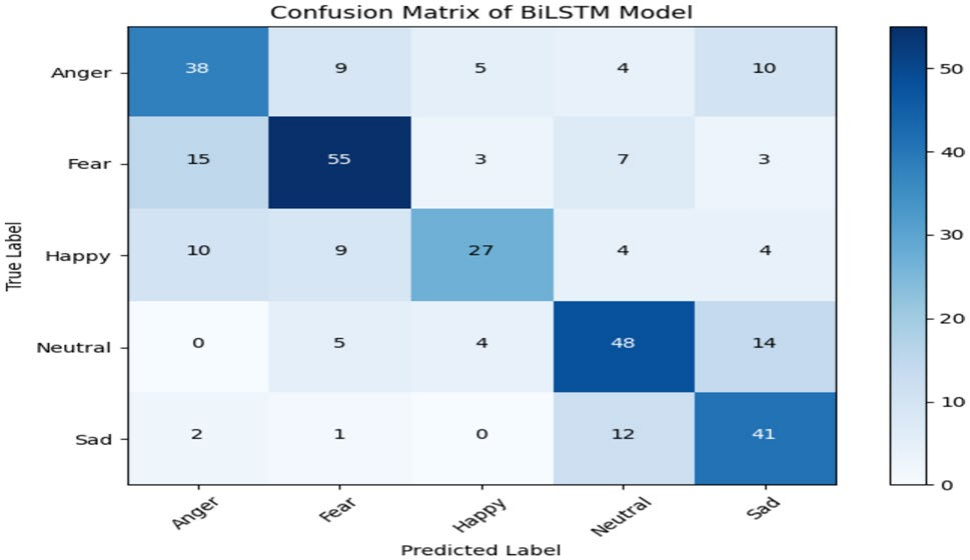
Confusion matrix of the BiLSTM model highlighting improved detection for Sad and Fear emotions.

**Fig. 11:**
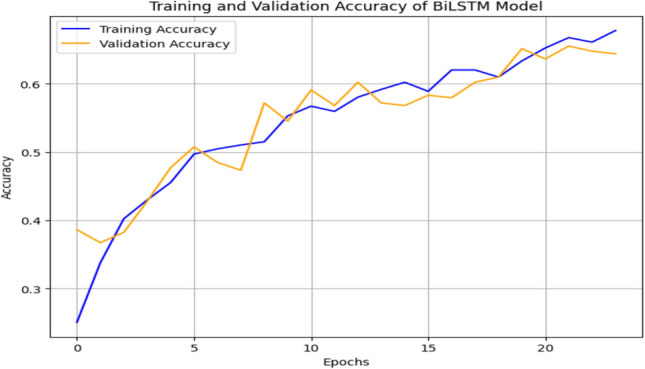
Training and validation accuracy of BiLSTM model.

**Fig. 12:**
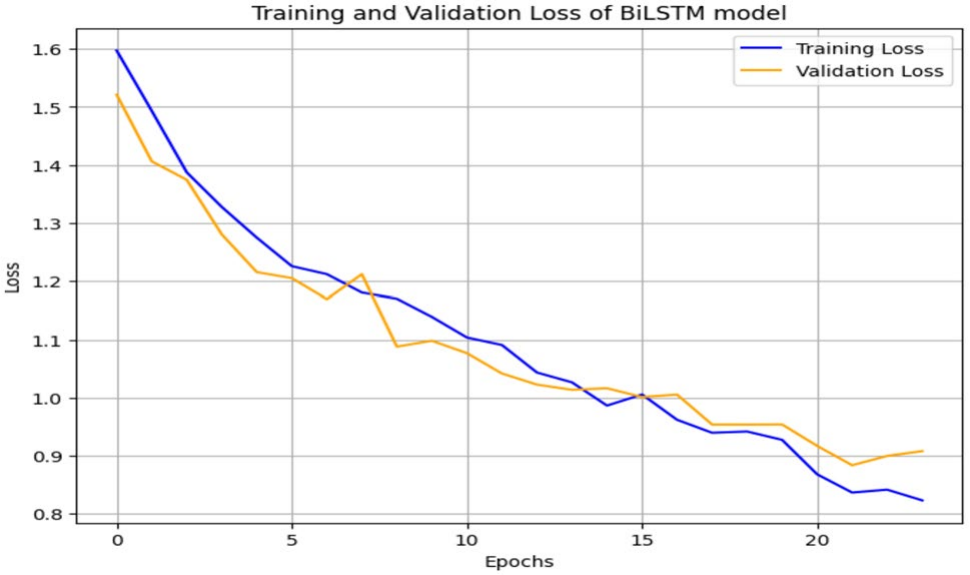
Training and validation loss of BiLSTM model.

The BiLSTM model enhanced the accuracy, achieving 63.33%. It was better at recognizing Sadness and Fear emotions but still failed to distinguish between happy emotions clearly. The confusion matrix in Fig. 10 indicated mistakes between Fear and Anger, and Neutral and Sad emotions. The training and validation curves, shown in Figs. 11 and 12, indicated steady learning.

#### GRU model

**Fig. 13:**
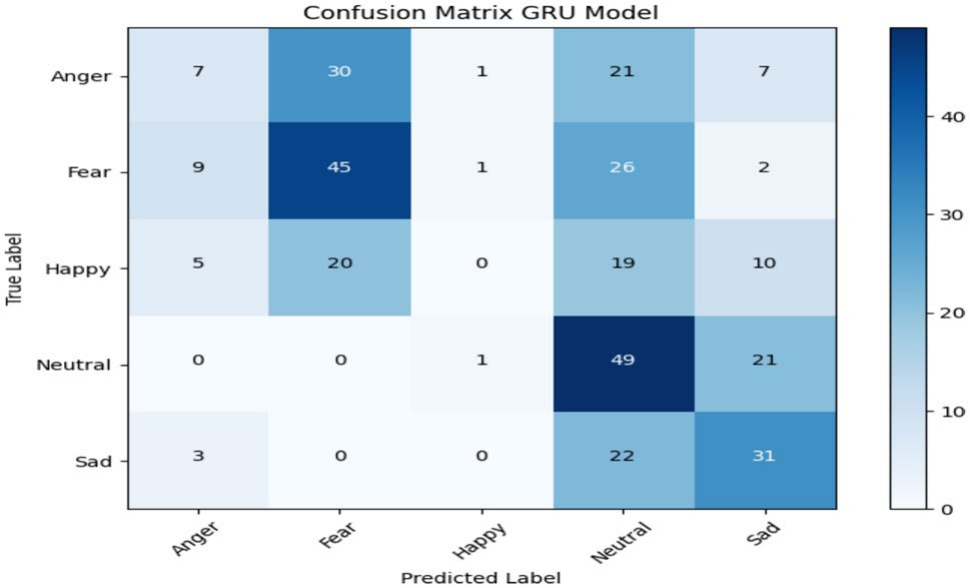
Confusion matrix of the GRU model showing poor recognition of Anger and Happy.

**Fig. 14:**
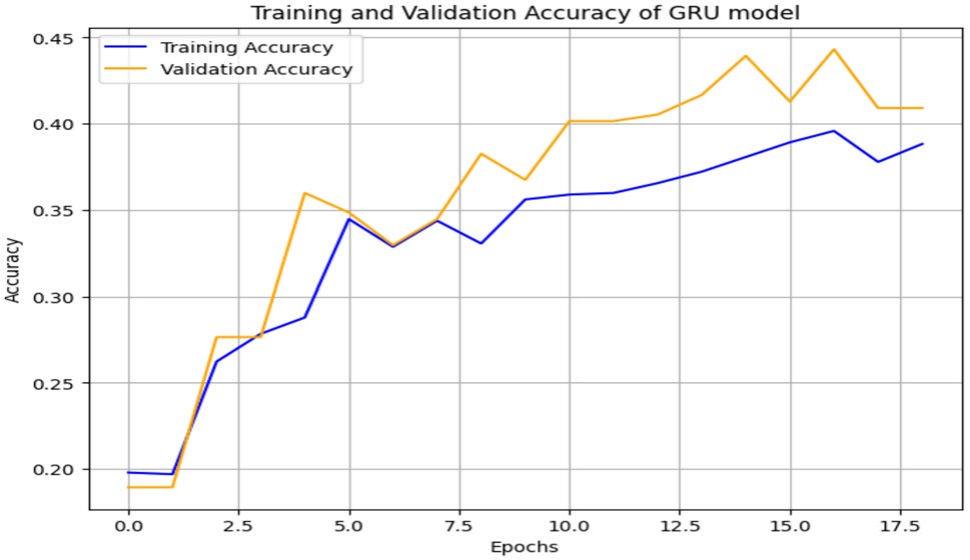
Training and validation accuracy of GRU model.

**Fig. 15:**
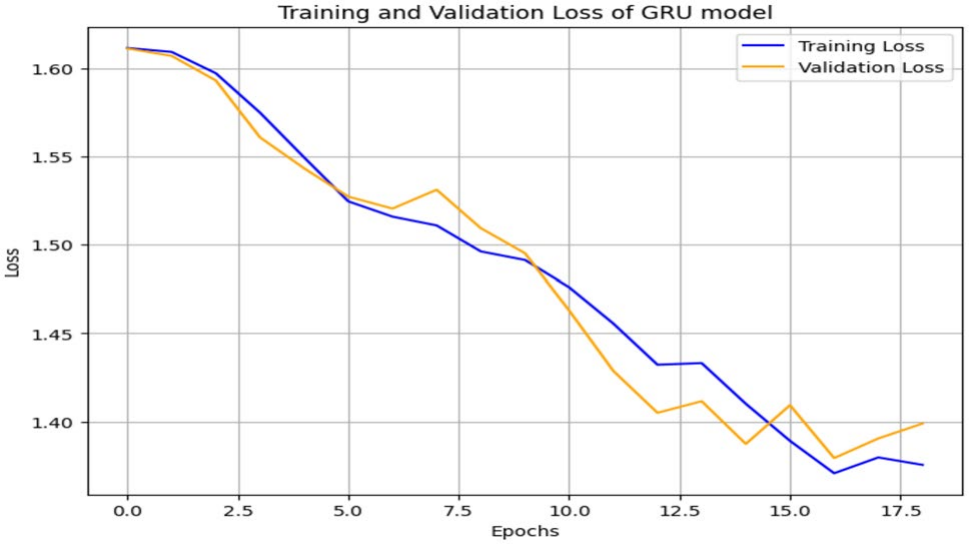
Training and validation loss of GRU model.

The performance of the GRU model was found to be the lowest, with 40% accuracy. The model struggled to recognize Anger and was not able to recognize the emotions of Happy. The confusion matrix in Fig. 13 indicated a high level of confusion. The training curves, shown in Fig. 14 and 15, indicated overfitting.

#### CNN model

**Fig. 16:**
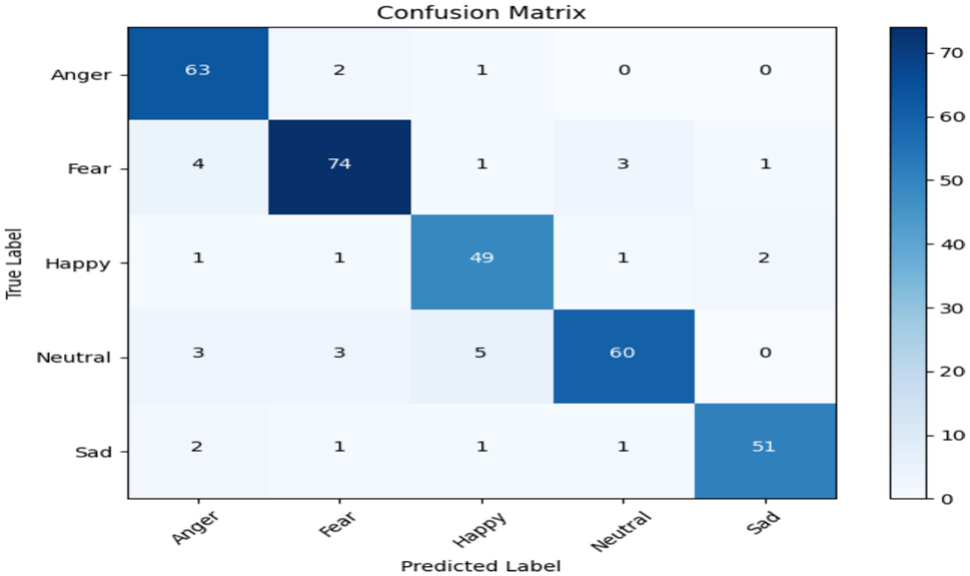
Confusion matrix of the CNN model demonstrating strong classification across all emotions.

**Fig. 17:**
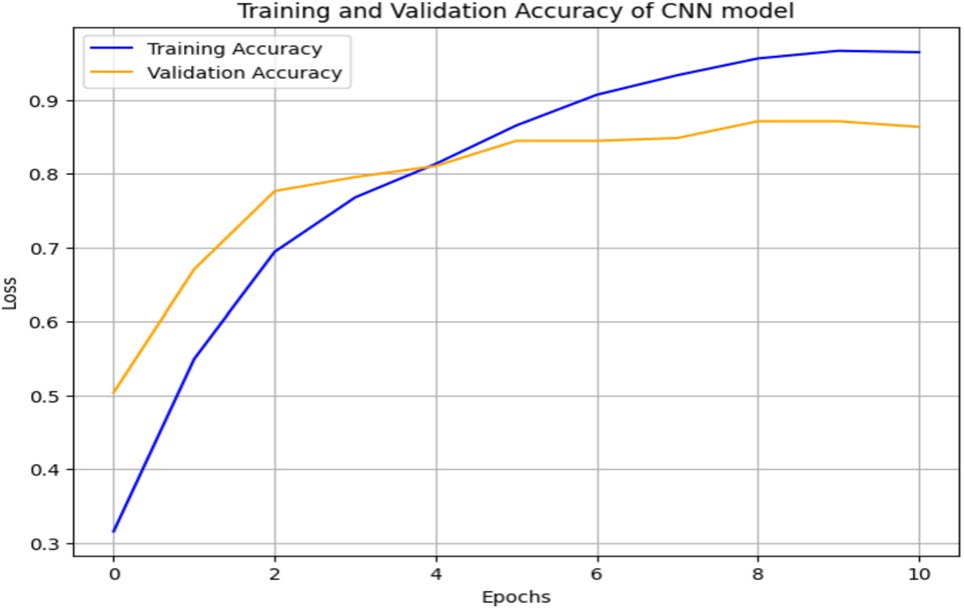
Training and validation accuracy of CNN model.

**Fig. 18:**
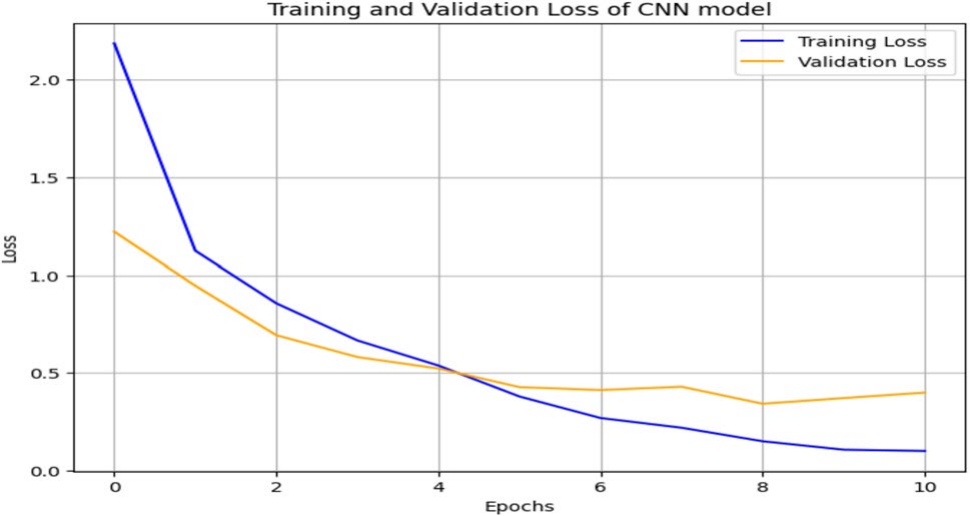
Training and validation loss of CNN model.

The proposed CNN model with both local and spectrogram features recorded the highest accuracy at 90%, with high precision, recall, and F1-score values. The confusion matrix in Fig. 16 revealed some errors, which were mainly for similar emotions. The training and validation data plots, shown in Figs. 17 and 18 indicate fast convergence with little signs of overfitting.

## Discussion of results

The experimental findings validate the efficacy of the proposed method for Amharic speech emotion recognition. The results highlight several important findings regarding the impact of preprocessing, feature integration, and model selection on overall system performance.

The proposed noise reduction method works well, which shows that preprocessing is an important step when working with real-world Amharic speech data, which is often affected by noise from the environment. Using a combination of noise reduction methods improved the quality of the signal and helped better represent the features, which in turn improved the performance of the classification. This finding is consistent with previous studies that emphasize the importance of preprocessing in speech emotion recognition^21,22^.

Feature integration played a critical role in making the model work better. By using both spectrogram-based features and local acoustic features, the model was able to capture both global and fine-grained local emotional characteristics. This hybrid feature fusion method helped make classification more accurate and robust, which backs up earlier research that shows feature fusion is a key factor in getting reliable SER performance^21,22^.

The CNN model consistently outperformed recurrent models including LSTM, BiLSTM, and GRU. The CNN model’s 90% accuracy is due to its ability to learn spatial patterns from spectrogram representations very well. Spectrograms contain both time and frequency information, which enables CNNs to perform hierarchical feature learning to better tell the difference between different emotional states. On the other hand, recurrent models mainly look at temporal dependencies and might not be able to effectively capture discriminative spectral features, which can lead to lower performance^16,18^. The performance gap between CNN and recurrent models is thus justified by differences in feature representation capabilities. Additionally, all models were trained under uniform experimental conditions, guaranteeing a fair comparison and not influence from variations in preprocessing or training configurations.

The confusion matrices showed that recurrent models had trouble telling the difference between emotional classes that were very similar, especially Neutral and Sad emotions. This shows that they can’t capture small changes in emotions very well. The GRU model, in particular, showed signs of overfitting, which made its lower performance.

**Fig. 19:**
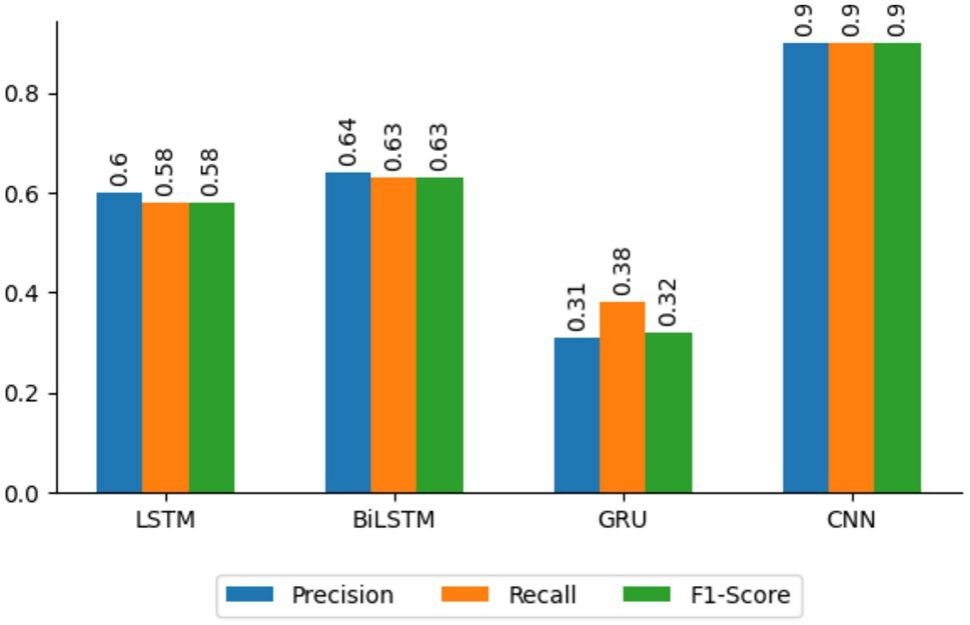
Comparison of accuracy, precision, recall, and F1-score among LSTM, BiLSTM, GRU, and CNN models.

The proposed CNN-based framework’s strong performance also shows that it is good for recognizing emotions in speech in low-resource languages like Amharic, where there are problems like not having enough annotated data and a lot of phonetic variation^29^. Combining advanced preprocessing methods, hybrid feature extraction, and CNN-based learning creates a strong and useful framework for recognizing emotions. Figure 19 further demonstrates that the CNN model outperforms all compared models in terms of accuracy, precision, recall, and F1-score when comparing the performance of CNN, LSTM, BiLSTM, and GRU models. These findings validate the effectiveness of the proposed methodology and establish a robust basis for subsequent research in low-resource language contexts.

## Conclusion and recommendations

In this paper, a CNN-based model for recognizing emotions from Amharic speech has been proposed. The performance of the proposed system was assessed through experiments conducted on a dataset comprising 1650 utterances of Amharic speech samples, where each speech sample lasted three seconds; of which, 1320 samples were considered as training set while 330 as the test set. Five categories of emotions were defined in the annotated corpus, namely anger, fear, happy, neutral, and sad. Our method incorporates global spectro-temporal information in the form of time–frequency spectrograms and local features in the form of MFCC, chroma, ZCR, pitch, and energy. This integration enables the CNN to efficiently extract global-Spectro temporal patterns, as well as local prosody information in the speech signals. Moreover, incorporating advanced pre-processing strategies, such as spectral subtraction combined with wavelet denoising, helps improve the resilience of the proposed CNN-based model to noise.

Experimental results show that the proposed CNN model can achieve an accuracy rate of 90% in classifying emotions with an equal level of precision, recall, and F1 score in all emotion categories. Compared to other neural networks, including LSTM, BiLSTM, and GRU, the proposed model performs significantly better. This demonstrates the effectiveness of combining different types of features as well as proper preprocessing techniques in speech emotion recognition, especially in low-resource languages. However, this study has several limitations. Firstly, the number of samples in the training and testing sets is small, thus limiting the generalizability of the results. While using speaker-independent data splitting, k-fold validation has not been considered in the current investigation. Furthermore, multimodal analysis has not been performed, since the current experiment only uses speech features. These limitations can be addressed by a more extensive database of speakers under different conditions, utilizing cross-validation methodologies for better assessments, and designing hybrid models that combine CNNs with recurrent neural networks or Transformer architectures. In addition, the use of multimodal inputs and the design of a lightweight model for real-time implementation are suggested for future work to enhance the practicality of the system.

In conclusion, this study shows that combining spectral and local acoustic attributes using a CNN-based system is a viable solution for detecting speech emotions in Amharic. This study makes contributions to the advancement of emotion detection for low-resource languages.

## Data Availability

The dataset generated during the current study is available from the corresponding author upon reasonable request.
